# Empirical evidence that metabolic theory describes the temperature dependency of within-host parasite dynamics

**DOI:** 10.1371/journal.pbio.2004608

**Published:** 2018-02-07

**Authors:** Devin Kirk, Natalie Jones, Stephanie Peacock, Jessica Phillips, Péter K. Molnár, Martin Krkošek, Pepijn Luijckx

**Affiliations:** 1 Department of Ecology and Evolutionary Biology, University of Toronto, Toronto, Ontario, Canada; 2 Department of Ecology, Behavior and Evolution, University of California San Diego, La Jolla, California, United States of America; 3 Department of Biological Sciences, University of Toronto Scarborough, Toronto, Ontario, Canada; Imperial College London, United Kingdom

## Abstract

The complexity of host–parasite interactions makes it difficult to predict how host–parasite systems will respond to climate change. In particular, host and parasite traits such as survival and virulence may have distinct temperature dependencies that must be integrated into models of disease dynamics. Using experimental data from *Daphnia magna* and a microsporidian parasite, we fitted a mechanistic model of the within-host parasite population dynamics. Model parameters comprising host aging and mortality, as well as parasite growth, virulence, and equilibrium abundance, were specified by relationships arising from the metabolic theory of ecology. The model effectively predicts host survival, parasite growth, and the cost of infection across temperature while using less than half the parameters compared to modeling temperatures discretely. Our results serve as a proof of concept that linking simple metabolic models with a mechanistic host–parasite framework can be used to predict temperature responses of parasite population dynamics at the within-host level.

## Author summary

Host–parasite interactions are impacted by temperature, and climate change is altering the nature of these interactions. Measuring how a range of temperatures affects host and parasite traits and how this influences the outcome of infections is infeasible in most systems. The metabolic theory of ecology provides a powerful framework to predict biological rates in response to temperature. Using a *Daphnia*–parasite model system, we collected experimental data on host survival and parasite abundance across the host’s temperature range. We fitted thermal relationships based on the metabolic theory of ecology to separate host and parasite traits, including host mortality and aging as well as parasite growth and virulence. We then provide empirical evidence of the predictive power of linking these relationships in mechanistic within-host parasite models. This allows us to predict the outcome of individual infections continuously across a temperature gradient, as well as to gain a better understanding of the impact of temperature changes on disease dynamics. Due to its simplicity and generality, this framework could be a valuable approach for predicting the effects of climate change on infection outcomes for hosts and microparasites.

## Introduction

The effects of global environmental change on infectious disease dynamics have broad consequences that span human health [1], food security [2], and conservation [3]. Climate change–driven temperature changes will likely impact the nature of host–parasite interactions, as both host and parasite traits can be strongly affected by temperature [4]. However, the effects of environmental change on disease dynamics are difficult to predict, because temperature can have differing effects on host and parasite traits that together determine outcomes for disease spread and severity [5].

There is ample evidence that temperature affects the dynamics of host–parasite interactions, and these effects are especially profound for ectoparasites and endoparasites within ectothermic hosts, as environmental temperature directly determines the rate of physiological processes in these cases [6]. In freshwater systems, for example, increased temperature typically causes earlier and extended parasite transmission, faster parasite growth, and more generations within a given season (but also higher parasite mortality) [7], and it has also been shown to lead to decreased parasite loads in amphibians [8]. In addition, temperature also influences the traits that mediate the impact of infection on host survival, such as parasite development rate [9] and virulence [10], as well as the resistance [11] and tolerance [12] of hosts to parasites. Each of these traits may have distinct temperature responses, and it is the antagonistic and synergistic interactions among the temperature responses of host and parasite traits that determine the net consequences of temperature changes on disease dynamics [13].

Temperature can affect hosts and parasites asymmetrically, leading to complex and nonintuitive infection outcomes. If traits such as host resistance and parasite growth respond similarly to temperature changes, these responses may trade off, resulting in no observed effect of temperature on parasite abundance [14]. However, if the strength of the temperature dependence of the host and parasite responses differs, warming may favor either the host or the parasite [14]. For example, higher temperatures increase host penetration but decrease successful encystment for the parasitic trematode *Ribeiroia ondatrae*, resulting in the greatest amount of parasite-induced malformations in its amphibian host at an intermediate temperature [15]. These types of dynamics are unlikely to be captured in empirical studies that expose individuals to a limited thermal range or in theoretical models that do not account for distinct temperature responses for host and parasite traits.

It has been suggested that the thermal dependencies of basic host and parasite traits (e.g., development, survival, movement) could be described by simple formulae arising from first metabolic principles outlined in the metabolic theory of ecology (MTE). These relationships could inform classical models of host–parasite dynamics to estimate the net effect of temperature changes on disease prevalence and severity [16–17]. However, to date, there is limited empirical evidence that this approach can effectively predict the consequences of environmental change for host–parasite population dynamics [13,16].

MTE posits that the thermal dependencies of biological processes across levels of biological organization can be deduced from the thermal dependency of the basic metabolic rate of individual organisms [18]. MTE further suggests that the Van’t Hoff–Arrhenius model can predict an organism’s metabolic rate and related processes based on temperature [18]. Indeed, the Van’t Hoff–Arrhenius model has been shown to adequately fit the rising region of many unimodal temperature response curves [19]. Multiple extensions of the Van’t Hoff–Arrhenius model exist [20] to also describe the low and high temperature threshold behaviors of (typically unimodal) thermal response curves [6], of which the Sharpe–Schoolfield model may be the most commonly used [21]. However, while the general functional form of the temperature dependencies of different traits (exponentially rising with temperature over the intermediate temperature range and unimodal over the entire temperature range) can usually be captured with these models, different traits of an individual may show distinct sensitivities to temperature due to differing suites of rate-limiting enzymes [22]. In addition, thermal sensitivities are likely to differ between host and parasite traits (e.g., host resistance versus parasite growth), further complicating forecasts of temperature effects on disease, as traits may interact in an exponential, multiplicative, or additive manner in models of the host–parasite dynamics.

In this study, we fitted a mathematical model to experimental data on *Daphnia magna* and its natural microsporidian parasite *Ordospora colligata* to examine the effects of temperature on host and parasite traits, their interactions, and the within-host parasite population dynamics. Over nine temperatures that span the thermal range of *D*. *magna*, we quantified host lifespan for both exposed and unexposed *D*. *magna* and measured parasite abundance at death. We used these data to fit a model for the survival of unexposed hosts (*U*), exposed hosts (*E*), and within-host parasite abundance (*P*) given by

$$\frac{dU}{dt}=-\beta_{U}\;\mu^{\beta_{U}}\;t^{\beta_{U}-1}\;U$$ (1)

$$\frac{dE}{dt}=-\;\beta_{E}\;{\left(\mu +\alpha P\right)}^{\beta_{E}}\;t^{\beta_{E}-1}\;E$$ (2)

$$\frac{dP}{dt}=rP(1-\frac{P}{\theta }),$$ (3)

where natural host mortality is modeled by a Weibull distribution with mortality rate *μ* and aging parameter *β*. If *β* = 1, mortality is constant through time. If *β* < 1, mortality decreases over time, and if *β* > 1, mortality increases over time. The parasite population growth rate is *r*, the per parasite virulence additively affecting host survival is *α*, and parasite equilibrium abundance within hosts is *θ*. In our model, *θ* is determined by both host and parasite processes and may therefore show positive or negative temperature dependence depending on whether host resistance or parasite processes are more strongly temperature dependent [13]. The host and parasite traits represented by the model parameters were first fitted separately for each temperature treatment and then also to all temperatures simultaneously using a global model that represented each trait using MTE formulations.

The MTE has been a major focus of ecological studies since its inception about two decades ago. Numerous meta-analyses have tested and refined MTE’s predictions across broad interspecific datasets [19, 23–25]. More recently, MTE has been the focus of theoretical developments attempting to understand how temperature changes may affect the dynamics of interacting species, such as in predator–prey or host–macroparasite systems [16, 26–28]. We show that the thermal dependencies of host and parasite traits affecting within-host parasite dynamics and host survival are well described by variants of the Van’t Hoff–Arrhenius and Sharpe–Schoolfield models and that nesting these relationships in a within-host parasite population dynamics model captures the resulting thermal dependency of the cost of infection (i.e., the percent reduction in mean lifespan due to infection). Our results empirically demonstrate the predictive power of linking MTE-based thermal relationships for host and parasite traits in mechanistic within-host parasite models to understand the impacts of temperature changes on disease. Because of its simplicity and generality, this framework provides a valuable approach for predicting the outcomes and consequences of infections for hosts and parasites in light of climate change.

## Results

### Data

**Fig 1:**
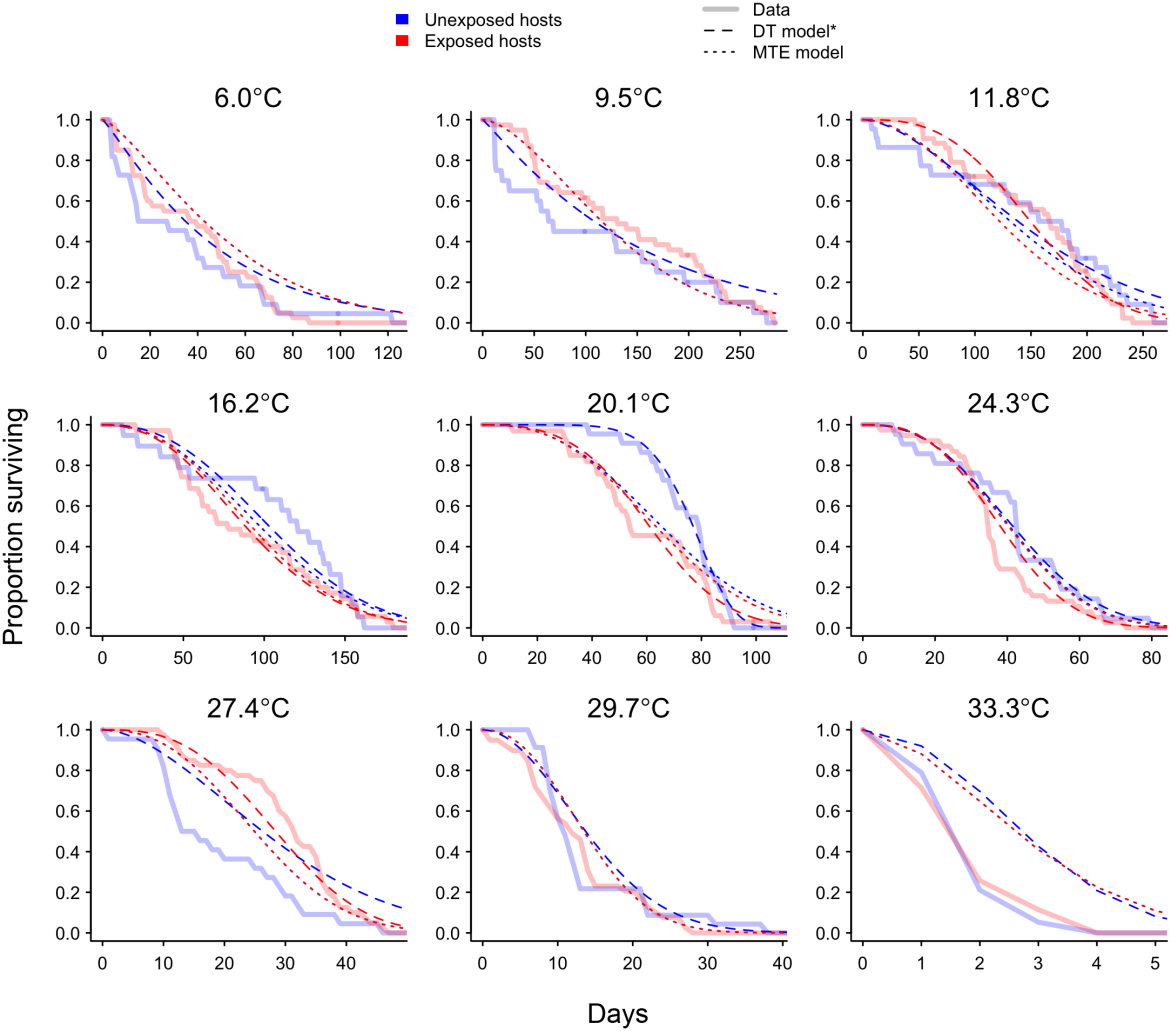
Solid lines show the proportion of unexposed (*n* = 195; blue) and exposed (*n* = 355; red) individuals surviving over the course of the 285-d experiment. Model fits are shown by the dashed (DT) and dotted (MTE) lines. The DT model does not make predictions for exposed individuals at 6.0 °C, 9.5 °C, 29.7 °C, or 33.3 °C due to convergence and estimability issues for some of the parasite-related parameters. The data used to make this figure can be found in S1 Data. DT, discrete temperature; MTE, metabolic theory of ecology.

**Fig 2:**
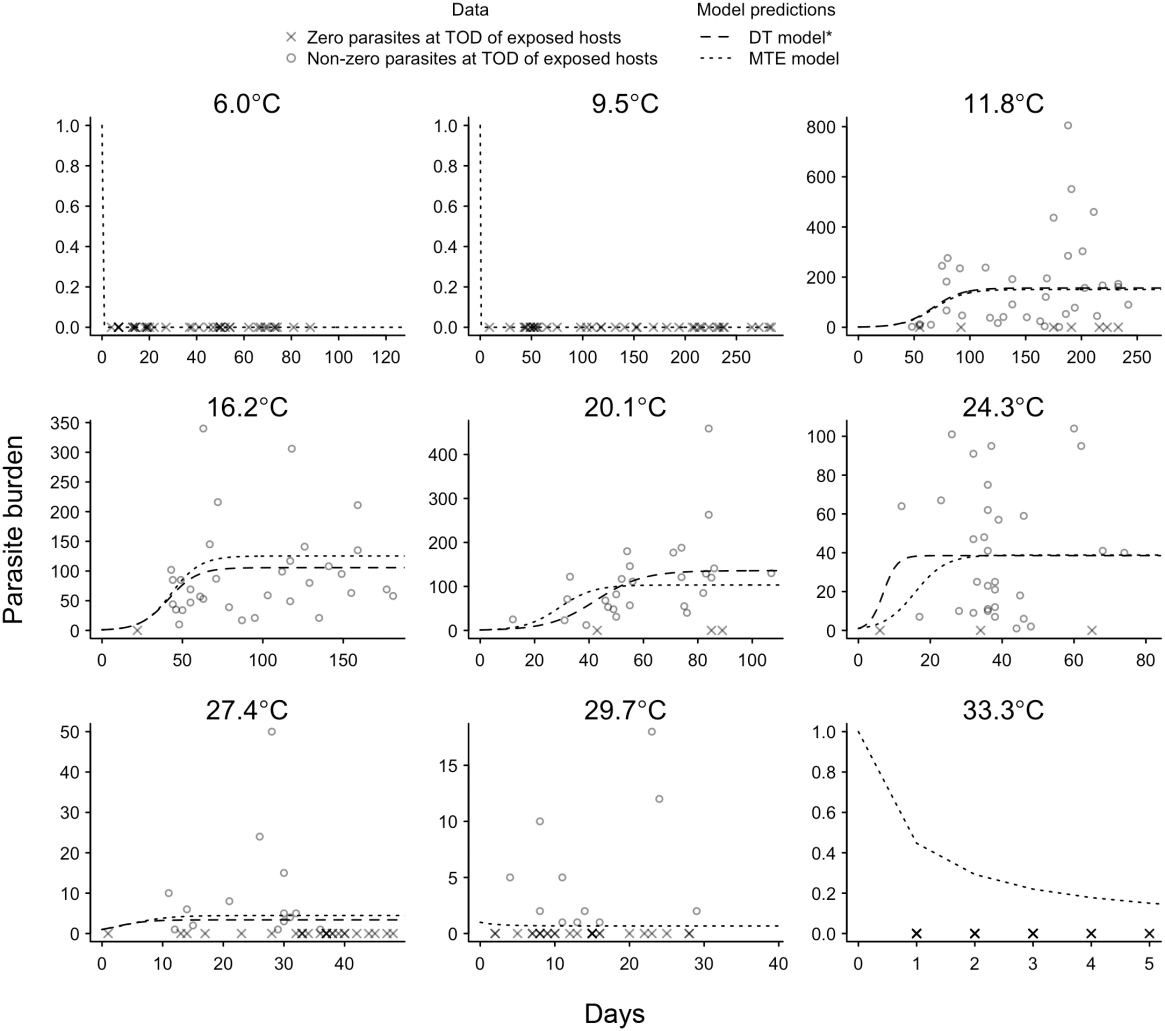
The number of parasites per exposed individual at TOD. Circles show nonzero parasite loads at TOD, while crosses represent exposed individuals who had zero parasites at TOD. Model fits are shown by the dashed (DT) and dotted (MTE) lines. Parasites were never observed at 6.0 °C, 9.5 °C, or 33.3 °C. The DT model does not make predictions for exposed individuals at 6.0 °C, 9.5 °C, 29.7 °C, or 33.3 °C due to convergence and estimability issues for some of the parasite-related parameters. The data used to make this figure can be found in S1 Data. DT, discrete temperature; MTE, metabolic theory of ecology; TOD, time of death.

We obtained survival and infection intensity data on 550 individuals across nine temperature treatments (6.0 °C–33.3 °C). None of the exposed individuals at 6.0 °C, 9.5 °C, or 33.3 °C became infected, while infection prevalence among exposed individuals at intermediate temperatures ranged from 28% to 97% (Table 1). Since the fate of all individuals is known (uncensored survival data), we report mean lifespan, which was greatest at 11.8 °C for both unexposed and exposed individuals (Table 1, Fig 1). Infection intensity at death was highly variable within and across temperatures, ranging from 1 to 805 spore clusters, with peak mean infection intensity occurring at 11.8 °C (Table 1, Fig 2). We also collected data on rate of host offspring production, which clearly indicate a temperature effect and perhaps a small effect of the parasite on host reproduction (S6 Fig, S6 Table). However, the host reproduction components of host–parasite population dynamics need to be analyzed using a model for disease spread at the host population level with MTE submodels for temperature effects, an approach that is beyond the scope of the current study.

**Table 1:** Summary of experimental data. Infection % is the percent of exposed individuals that were infected upon inspection at death. Infection intensity is the number of spore clusters in an individual at death and was calculated using only individuals who had a nonzero parasite load. Mean survival time was calculated for the entire treatment sample, including exposed individuals with parasite load zero.

Temp(°C)	Sample Size		Infection %	Infection Intensity (mean ± s.e.)	Mean Survival Time (d)	
	Unexposed	Exposed			Unexp.	Exposed
6.0	22	40	0.0	NA	33.9	38.5
9.5	20	39	0.0	NA	107.2	139.9
11.8	22	43	83.7	162.5 ± 29.5	147.7	152.2
16.2	19	35	97.1	93.8 ± 13.4	107.8	92.5
20.1	22	33	90.0	113.1 ± 17.6	75.6	61.1
24.3	21	38	91.2	41.1 ± 5.9	43.2	38.3
27.4	22	41	34.1	9.6 ± 3.5	20.5	30.4
29.7	23	39	28.2	5.4 ± 1.7	15.0	13.8
33.3	24	47	0.0	NA	2.6	2.6

### Model fitting

We modeled the within-host dynamics using three differential equations for the changes in the numbers of unexposed individuals (*U*, Eq 1), exposed individuals (*E*, Eq 2), and parasites within each infected individual (*P*, Eq 3) over time. We fitted this model to the experimental data and estimated host and parasite parameters across temperatures using two different approaches. First, we estimated the model parameters independently at each temperature (discrete temperature [DT] model). Second, we described each of these parameters continuously across temperature using variants of the Van’t Hoff–Arrhenius and Sharpe–Schoolfield models (S1 Table). We chose the MTE function for each parameter based on how the DT estimates appeared to change qualitatively with temperature (initially, we aimed for a model selection approach fitting numerous MTE functions for each parameter, but this quickly became infeasible due to the large number of possible submodel combinations and the computational time it took to fit each model). We estimated the hyperparameters (e.g., activation energies that drive temperature sensitivity at intermediate ranges, temperature thresholds, inactivation energies that determine how quickly trait performances fall beyond thresholds) associated with these MTE models, allowing us to predict the host and parasite parameters and subsequently host survival and parasite intensity continuously across temperature.

**Fig 3:**
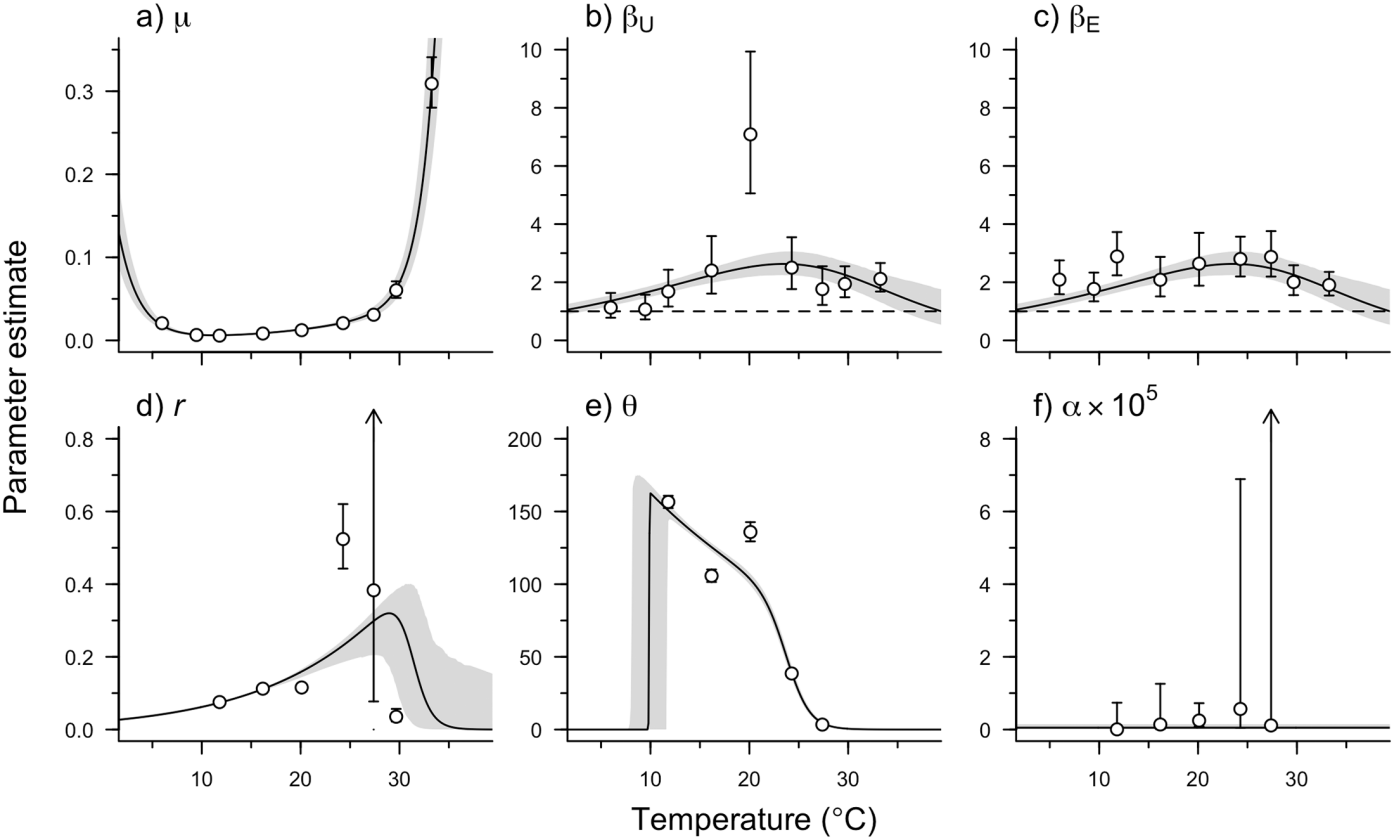
Maximum likelihood estimates ± 95% confidence intervals for the DT model (points and vertical bars) and fitted MTE functions ± 95% confidence intervals (lines and shaded region) for host–parasite model parameters: a) natural host mortality *μ*, b) natural mortality shape parameter for unexposed individuals *β*_U_, c) natural mortality shape parameter for exposed individuals *β*_E_, d) parasite growth rate *r*, e) parasite equilibrium abundance *θ*, and f) parasite virulence *α*. *β*_*U*_ (unexposed data) and *β*_*E*_ (exposed data) were estimated separately for DT estimates, but shared the same MTE function (Sharpe–Schoolfield with upper threshold). The 95% confidence interval on MTE model predictions was calculated as the 95% quantiles of 1,000 Monte Carlo samples of the host–parasite model parameters; in each Monte Carlo sample, the associated hyperparameters were chosen from a normal distribution with mean and SE of the MLE. Parameters that were not estimable are not shown (see S1 Text for details). DT, discrete temperature; MTE, metabolic theory of ecology.

The DT estimates for the mean mortality rate of hosts (*μ*), as well as for the Weibull shape parameters (*β*_i_) that describe age-related changes in these mortality rates, showed temperature dependence. As expected, mean mortality was lowest just above the host’s lower thermal threshold, increased with increasing temperature over most of the thermal niche, and rose steeply to infinity (indicating immediate mortality here) at low and high temperature extremes (*μ*; Fig 3). The shape parameters *β*_i_ were highest at intermediate temperatures and greater than one for both unexposed (*β*_U_) and exposed hosts (*β*_E_; Fig 3), except at the lowest temperatures, where the confidence interval for *β*_U_ overlapped one. This indicates that mortality rate increases as the host ages and that the aging process is strongest at the intermediate temperatures, perhaps because individuals had high mortality at temperature extremes and thus did not have an opportunity to age. There was no consistent difference in *β* between unexposed and exposed hosts, although we note that *β* was significantly higher for unexposed hosts at 20.1 °C, indicating delayed mortality for unexposed individuals at this temperature.

**Fig 4:**
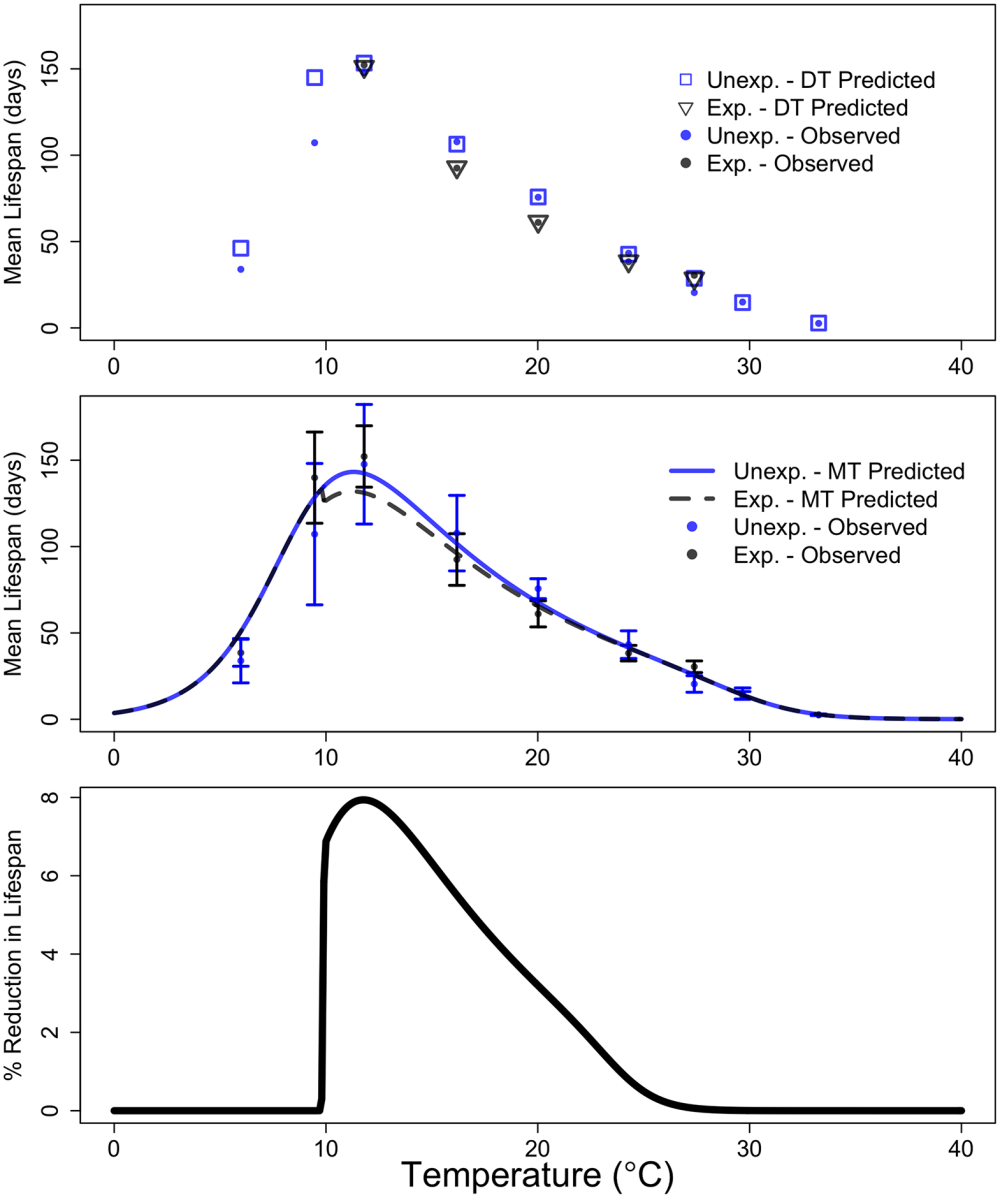
Observed (filled circles, ± 95% CI shown in b) and predicted mean lifespans of hosts from DT (a: unfilled squares and triangles) and MTE (b: solid and dotted lines) models, as well as the MTE-predicted percentage cost of infection across the temperature range (c). The DT model can only predict mean lifespan at the nine temperatures where we have observed data, whereas the MTE model is able to make predictions across any temperature range of interest. The data used to make this figure can be found in S1 Data. DT, discrete temperature; MTE, metabolic theory of ecology.

Parasite population growth rate (*r*) increased from 11.8 °C to 24.3 °C before decreasing at higher temperatures (Fig 3). Similarly, equilibrium parasite abundance (θ) decreased sharply at temperatures above approximately 20 °C (Fig 3). Parasite virulence *α* also appeared to increase with temperature, but high uncertainty in the estimates of *α* obscured any clear thermal relationship (Fig 3). Combining these DT estimates of temperature dependencies within the host–parasite model (Eqs 1–3) accurately predicted the observed lifespan of exposed and unexposed individuals across most of the temperature range (Fig 4a). Parasite parameters were difficult to estimate at the extreme low and high temperatures because no parasites were found in hosts at 6.0 °C and 9.5 °C and hosts did not survive long enough to allow for within-host parasite growth at 29.7 °C and 33.3 °C (Figs 1 and 2). Thus, at these four temperatures, we were not able to reliably estimate *r*, θ, and *α* using the DT approach because our model-fitting algorithm did not converge or parameters were found to be nonestimable given the available data (see S1 Text for details).

In the MTE-based approach to estimating the temperature dependencies of model parameters, we used a single *β* parameter for *β*_*U*_ and *β*_*E*_, as DT estimates did not reveal any consistent differences. All hyperparameters were estimable for the MTE approach, with the exception of the upper inactivation energy for *β* and the lower inactivation energy and threshold for *θ* (Table 2, S4 and S5 Figs). The activation energies of *β*, *μ*, *r*, and *θ* were all within the typical 0.2–1.2 eV range (Table 2) [19] but differed significantly from each other. The activation energy associated with parasite population growth (0.669 ± 0.0278, Table 2), for example, was positive, while the activation energy of equilibrium abundance was negative (−0.295 ± 0.0232, Table 2), reflecting contrasting temperature dependencies for these parameters at intermediate temperatures. By contrast, parasite virulence (*α*)—estimated as the daily per parasite-induced host mortality—was not only very small (indicating that this parasite isolate is relatively benign in this host genotype), but also the only model parameter that was modeled as temperature independent due to the lack of a clear pattern in the DT estimates.

**Table 2:** MLEs and SE for the hyperparameters associated with metabolic models for each of the host–parasite parameters (S1 Table) from 25 clones of the data. The MCMC chains converged for all parameters ($\hat{R}<1.1$), but some parameters were not estimable and are highlighted in grey.

Parameter	Hyperparameter	MLE	SE	$\hat{R}$	Estimable?
Mean mortality rate (*μ*)	μ_0_	0.00751	0.000343	1.01	Yes
	*E*_*μ*_	0.801	0.0404	1.01	Yes
	*E*_*Hμ*_	4.24	0.278	1.00	Yes
	*T*_*Lμ*_	8.96	0.234	1.01	Yes
	*T*_*Hμ*_	30.2	0.302	1.01	Yes
Shape parameter in Weibull (*β*_U_, *β*_E_)	*β*_0_	2.28	0.107	1.00	Yes
	*E*_*β*_	0.391	0.0363	1.00	Yes
	*E*_*Hβ*_	1.47	0.225	1.00	Yes
	*T*_*Hβ*_	28.8	0.500	1.00	No
Parasite growth rate (*r*)	*r*_0_	0.0998	0.00125	1.00	Yes
	*E*_*r*_	0.669	0.0278	1.00	Yes
	*E*_*Hr*_	8.83	4.38	1.00	Yes
	*T*_*Hr*_	31.2	1.14	1.00	Yes
Parasite equilibrium abundance (*θ*)	*θ*_0_	131	1.41	1.00	Yes
	*E*_*θ*_	-0.295	0.0232	1.00	Yes
	*E*_*Lθ*_	357	1320	1.01	No
	*E*_*Hθ*_	6.32	0.189	1.00	Yes
	*T*_*Lθ*_	9.87	0.896	1.00	No
	*T*_*Hθ*_	23.9	0.0985	1.00	Yes
Parasite virulence (*α*)	*α*_0_	5.12E-06	4.40E-06	1.00	Yes

**Abbreviations:** MCMC, Markov chain Monte Carlo; MLE, maximum likelihood estimate.

Despite having less than half as many parameters, the MTE model (20 free parameters) predicted host survival almost as well as the DT model (54 free parameters) (Figs 1, 2 and 4). The notable exception was at 20.1 °C, where the survival of unexposed hosts was initially very high and the DT model was flexible enough to capture this while the MTE model did not (Fig 1). The parameterized MTE model appropriately predicted differences in mean survival between unexposed and exposed individuals at 16.2 °C and 20.1 °C (Fig 4) and accurately predicted that there will be a very small cost of infection at the lower and higher temperatures. The only temperature at which the MTE model did not predict the cost of infection well was 11.8 °C, where exposed individuals did not appear to suffer costs in survival despite a cost being predicted by the MTE model (Fig 4). Infected individuals at this temperature did have high infection loads on average (Table 1, Fig 2), suggesting decreased parasite virulence at approximately 12 °C. This is supported by the DT estimate of virulence at 11.8 °C, which is more than an order of magnitude smaller than the next-smallest DT estimate of virulence at 16.2 °C (S4 Table). However, as mentioned above, the relatively large standard error associated with the DT estimates of virulence obscured any clear pattern across temperature, which is why we modeled virulence as temperature independent.

## Discussion

Accurate predictions of how climate change will alter host–parasite dynamics and disease require models that successfully integrate distinct thermal dependencies for a range of host and parasite traits. Here, we analyzed experimental data of host survival and parasite burden across a temperature gradient using an MTE-based model of within-host parasite population dynamics to capture the thermal dependencies of host aging and mortality as well as of parasite virulence, population growth rate, and equilibrium abundance. Our results offer new insights into how the thermal dependence of these traits can affect within-host parasite dynamics and show that linking MTE models with mechanistic host–parasite models provides an effective method for predicting disease dynamics across a wide thermal range. Our modeling approach allows prediction of disease dynamics as a continuous variable of temperature throughout the host’s and parasite’s thermal range, efficiently interpolating beyond and between the discrete temperature treatments, and at a cost of less than half the parameters that would need to be estimated when modeling temperature discretely. More generally, our results reveal that temperature does not impact host and parasite traits equally or symmetrically, which will likely complicate forecasting efforts for how climate change will affect disease dynamics.

MTE proved capable of capturing how four traits of the host–parasite system change with temperature. The natural mortality rate of hosts (*μ*) was modeled using the Sharpe–Schoolfield function, and the fitted model aligns closely with the DT estimates of *μ* (Fig 3a). Although it was unsurprising that the model fit well at the high and low temperatures, as mortality often peaks at extreme temperatures [16,20,29], MTE also accurately described the increasing mortality rate with rising temperature throughout the intermediate temperature range. Our model for host survival used a Weibull distribution that allows the natural mortality rate to change over time according to a shape parameter *β*, which can be interpreted as a measure of aging [30]. Our results suggest that the rate of aging in *D*. *magna* is temperature dependent, a finding that has been shown in ectotherms before (e.g., [31]). Notably, the chemical processes that regulate aging have been thought to be temperature dependent for decades [32], and if we consider the aging process in a physiological framework, a mechanistic link between aging and temperature can be drawn [18].

The free radical theory of aging posits that oxidants such as O_2_^−^ and H_2_O_2_ play a key role in the aging of an organism [33–34]. Since these oxidants are inadvertently produced during aerobic metabolism [35], if metabolism scales with temperature as predicted by MTE, then so should the rate of aging. Under this framework, the rate of accumulation of oxidants and, therefore, the rate of change in the mortality hazard (i.e., aging) will scale with temperature. In addition to the free radical theory, there are several further physiological and evolutionary theories of aging that may be operating independently or in combination with other mechanisms [36]. Ultimately, many of these theories invoke physiological mechanisms, such as DNA mutations, protein damage, and waste accumulation [36]. This again suggests that in organisms where these mechanisms are believed to operate, the rate of aging should vary with temperature. Overall, the average rate of mortality will depend on both the rate of aging (*β*) and the mortality scale parameter (*μ*), both of which are temperature dependent but in different manners (Fig 3).

Virulence was the only model parameter that seemed unaffected by temperature. Although defined here as the daily per parasite–induced host mortality, virulence can be viewed as an interaction term between the host and the parasite. The temperature dependency of virulence arises from a product of two temperature-dependent functions: one describing the damage a parasite does (e.g., cell lysis rate) and one describing the host’s counteracting responses (e.g., immune function) [13]. The observed temperature independence of virulence thus indicates that the temperature dependencies of the counteracting host and parasite processes determining virulence may have cancelled each other out in our system. In general, however, this need not be the case in other systems; for example, thermal mismatches between amphibian hosts and a fungal pathogen can significantly affect the host’s susceptibility to infection [37]. Furthermore, other within-host parasite traits whose thermal dependency is also determined by the interacting temperature dependencies of host and parasite metabolism do not need to cancel each other out fully either.

In our system, both the growth rate (*r*) and equilibrium abundance (*θ*) of parasites are likely influenced by host and parasite processes. Increased parasite metabolism should result in faster spore replication, which in turn will lead to the acceleration of host cell lysis and subsequently higher transmission between cells and increased growth rate. MTE suggests that the thermal dependencies of these processes can be captured using the Sharpe–Schoolfield model [38]. The thermal dependencies of *r* and *θ*, thus, should also (at least approximately) follow the Sharpe–Schoolfield equation [13], as is indeed observed (Fig 3). Interestingly, *r* increases with temperature while *θ* decreases with temperature throughout the intermediate temperature range (Fig 3). As with virulence, these temperature dependencies likely reflect counteracting temperature dependencies of host and parasite processes cancelling each other out to some degree [13], though in this case, not entirely.

The lower temperature threshold and inactivation energy for *θ* were not estimable (Table 2), but this is simply because no successful infections (nonzero parasite abundances) occurred below 11.8 °C (Fig 2). We suspect that the impaired infectivity below 11.8 °C may be due to mechanical failure of the spore’s infection apparatus at low temperatures, but the exact threshold temperature beneath which infection becomes impossible is unknown. We note that the most cogent approach would be to independently model the host processes that determine the parasite population growth rate and equilibrium abundance (e.g., number of available gut epithelial cells, immunity, body size [39,40]) as well as the parasite processes that counteract the host processes (e.g., parasite’s ability to infect a cell) and then to link these models mechanistically [13]. Although our experimental design could not decouple the host and parasite processes that determine parasite growth rates and equilibrium abundance, the net effect of these processes on host survival could nevertheless be predicted (Fig 4).

As expected, discrete temperature estimates were generally better at capturing the survival dynamics of unexposed and exposed individuals compared to estimates predicted from MTE (Figs 1, 2, 4): a model with more parameters will almost always fit the data better. However, while the discrete temperature model appears to be a better fit, it may not have high predictive power or provide general insight. Using the MTE-based submodels for the thermal dependencies of host and parasite traits, by contrast, required fewer parameters (20 compared to 54 in the DT model) and allows predicting survival and infection dynamics throughout the host’s thermal range by mechanistically interpolating and extrapolating model predictions relative to the structure of data inputs. However, although the MTE model had fewer parameters than the DT model, there were problems with estimability of three parameters associated with temperature thresholds (Table 2). These estimability problems may have resulted from structural identifiability problems in the model or, more likely, because we lacked data on parasite growth and survival at extreme temperatures. The nonestimability of these model parameters introduces higher uncertainty in the estimates of survival and the cost of infection near the upper and lower thermal limits of the host and parasite relative to the uncertainty in the intermediate temperature range.

The strong performance of the MTE model over most of the intermediate thermal range suggests that the MTE approach could be useful in predicting responses of infectious disease to climate change–induced temperature changes over most of the geographic range of a host–parasite combination. Problems with parameter estimability led to limited predictive power at the temperature extremes, but our results suggest satisfactory model performance for the majority of the temperature range. An among-host model of infectious disease dynamics could help to formally disentangle nonestimable parameters (e.g., [16–17]). In the case of the *D*. *magna–O*. *colligata* system, the limits to estimability may not impose practical limits on the utility of the modeling framework for predicting disease responses to climate change if the estimability issues only occur at the extreme temperatures that are rarely experienced by the host–parasite pair.

Here, we have focused on the within-host parasite population dynamics only, so we did not measure or model density-dependent drivers of the host dynamics or their influence on parasite transmission or host demographics. However, host density can affect parasite prevalence and transmission rates in natural communities (e.g., [41]), and expanding this work to include density dependence at the host population level could reveal interesting host–parasite interactions and temperature dependencies not captured here. For example, persistence of malaria in East Africa is affected by the interplay between host density and temperature [42]. Our approach of embedding MTE relationships within a system of differential equations describing within-host parasite dynamics could easily be expanded to models of the between-host microparasite dynamics, as has been done for macroparasites [16]. In our system, the between-host dynamics could be represented using a simple susceptible–infected model with environmental transmission, where the parameters composing the model include contact rate between hosts and parasites as well as the rate of shedding of parasites out of the host and parasite mortality in the environment. Such an approach would lead to the potential of nesting the within-host model described here within a between-host model, allowing for the characterization of disease dynamics across temperature for two intricately linked levels of biological organization [43]. The general capability of this approach will hinge on how well MTE can capture the additional demographic parameters involved in transmission and host population dynamics, which further empirical studies can help resolve.

Previous work has investigated the ability of MTE models to describe the temperature responses of an organism’s metabolism [23], as well as the responses of specific, distinct traits such as mortality and development [16,19]. In addition, theoretical papers have recently explored the multiple ways in which such traits can interact to determine population dyamics and among-species interactions [27–28]. Our findings build on this literature, demonstrating how differing thermal sensitivities of different traits interact in a model system to determine within-host parasite dynamics. While the *Daphnia*–parasite model system may be relatively simple, MTE principles are likely to hold in natural and more complex host–parasite systems [44]. Our study provides a proof of concept that MTE functions can accurately represent the thermal dependence of demographic parameters at the within-host level and that disease models based on these functions can capture empirical data across a wide thermal range. Application of similar principles to more complex systems could constitute a major step toward forecasting the epidemiological impacts of climate change.

The overall utility of combining host–parasite models with MTE in other systems will hinge on two factors. First, the population dynamics and interactions of hosts and parasites need to be described by an adequate model (Eqs 1–3 in our system), a problem of structural uncertainty that can be addressed by natural history knowledge and model selection statistics. Second, the temperature dependence of model parameters needs to be described, requiring knowledge of both the functional relationship between temperature and a parameter (e.g., the Sharpe–Schoolfield model) and estimates for each function’s hyperparameters (e.g., activation energies). Appropriate a priori choices for these relationships and parameters can initially be informed using MTE [13] and subsequently refined in empirical studies as done here. The multiple interacting temperature dependencies of host–parasite systems complicate forecast attempts, but explicitly formulating these dependencies in models based on first principles is a key step toward a predictive framework that is both generalizable and customizable across different transmission modes, species, and regions.

## Materials and methods

### Study species

The zooplankton species *D*. *magna* (order Cladocera) is a model organism widely used for testing and refining biological theories, including those concerning host–parasite interactions [45]. *O*. *colligata* is a natural microsporidian parasite of *D*. *magna* that infects the host’s gut epithelial cells [46]. After encysting, *O*. *colligata* replicates intracellularly, forming clusters of up to 64 spores that are released during cell lysis and subsequently either infect adjacent gut cells or are expelled into the water column, where they can infect new hosts. This parasite can have a high prevalence in natural populations and has low virulence compared to other *D*. *magna* parasites [45]. Both the host clone and parasite isolate were originally collected from the Tvärminne Archipelago in Finland.

### Experimental procedure

We exposed individual juvenile females of a single clone of *D*. *magna* to spores of *O*. *colligata* at nine different temperatures that encompass the full thermal tolerance range of *D*. *magna* (6.0 °C–33.3 °C, S5 Table). In total, we had 48 replicates for the parasite exposure treatment and 24 replicates for the parasite-free (control) treatments, each crossed by nine temperatures for a total of 648 *D*. *magna*. Prior to giving birth, mothers of the juveniles were kept in standardized conditions at approximately 20 °C to minimize maternal effects. When the juvenile *D*. *magna* were between two and four d old, they were randomly assigned to a temperature and parasite exposure treatment and transferred into individual mesocosms containing 80 ml Artificial *Daphnia* Medium (ADaM) [47] and 15 million batch-cultured algae (*Monoraphidium minutum*), where they acclimated for 48 h at their assigned temperature. Prior to applying any infection treatments, all individuals that died during the acclimatization period were replaced with backups that had been treated identically up to that point.

After acclimatization, we exposed 48 randomly selected individuals at each temperature to *O*. *colligata* by adding 1 ml spore solution containing approximately 28,000 spores to their mesocosm each day for four d for a total dose of approximately 112,000 spores. The remaining 24 unexposed individuals received 1 ml of a placebo each day for four d. On day 0 of the experiment, we fed each individual approximately 15 million algae; subsequently, for the remainder of the experiment, they were fed approximately 30 million algae three times per wk. Every seven d, each individual was transferred into a new mesocosm containing fresh ADaM and algae.

The experiment ran for 285 d. We followed individuals until their death so that we could determine lifespan, fecundity, and parasite abundance at time of death; the last individual died on day 285. We assessed host vitality daily by examining individuals for movement and collected data on host reproduction (S6 Fig, S6 Table) by counting and removing offspring twice per wk (S1 Text). Both exposed and unexposed individuals were immediately dissected upon death and observed using a 400x phase contrast microscope to determine the parasite load, defined as the number of spore clusters in a host at death. No unexposed individuals were infected, confirming that no contamination occurred. Deceased exposed individuals that we were unable to successfully dissect were placed in microcosms with uninfected juveniles, and juveniles were dissected after 14 d for infection status. Eight microcosms were found to contain infected juveniles, and in these cases, the primary individual was recorded as infected but with an unknown parasite load at death. Further details on temperature treatments, acclimatization, and experimental methods can be found in S1 Text.

### Model

We described the host–parasite dynamics using three differential equations for the changes in the numbers of unexposed individuals (Eq 1), exposed individuals (Eq 2), and parasites per infected individual (Eq 3) over time. To capture potential changes in the natural mortality rate of hosts with age [30], host survival was modeled using a two-parameter Weibull distribution that allows for a nonconstant hazard of dying. The scale parameter of this Weibull distribution (*μ*) is proportional to the mean mortality rate, and the shape parameter (*β*) controls how the mortality rate changes over time. If *β* = 1, mortality is constant over time and the constant hazard model described by the exponential distribution is recovered; however, if *β* < 1, mortality decreases over time, and if *β* > 1, mortality increases over time. We allowed *β* to differ between unexposed (β_U_) and exposed (β_E_) individuals because we anticipated that the progression of infection over the course of the experiment might influence how mortality changes over time. Infected individuals also suffered parasite-induced mortality, which we modeled as a linear increase per parasite in the scale parameter (i.e., mean mortality) at rate *α* [48]. Parasite population growth was modeled using a two-parameter logistic growth model, based on the maximum growth rate (*r*) and the equilibrium abundance (*θ*). Here, *θ* is interpreted as a stable equilibrium number of parasites resulting from interacting host and parasite processes, rather than the carrying capacity of parasites per host. The assumption of density dependence is supported by an inspection of the data that indicate a density-dependent constraint on upper levels of parasite abundance. A simple linear model of parasite growth provides a poor fit to the parasite abundance data whose time series, though variable, are clearly not concave up (Fig 2).

In order to understand the net effect of temperature on the host–parasite dynamics, we allowed each model parameter (*μ*, *β*, *α*, *r*, and *θ*) to be a function of temperature. First, we estimated parameters independently for each temperature (DT model). However, we were more interested in whether basic relationships from MTE could accurately describe the relationships between parameters and temperature. According to MTE, within intermediate temperatures of the organism’s thermal niche, rates will scale in proportion to the Boltzmann factor, exp(-*E*/*kT*), where *E* is the activation energy, *k* is Boltzmann’s constant (*k* = 8.62 × 10^−5^ eV K^−1^), and *T* is temperature in K [23]. The resulting equation for the rate at temperature *T*, with the Boltzmann factor standardized to a reference temperature of *T*_0_, is known as the Van’t Hoff–Arrhenius relation. To accommodate high or low temperature inactivation of metabolic enzymes, the Van’t Hoff–Arrhenius relation can be adapted to include upper and lower temperature bounds on rates, resulting in the Sharpe–Schoolfield function [21]. The Sharpe–Schoolfield relation is desirable because it captures the unimodal dependence of physiological rates on temperature and possesses the flexibility to include just the upper or lower bound, which we refer to as the modified Sharpe–Schoolfield model.

The MTE function for each parameter was chosen based on the qualitative temperature dependence of the DT estimates, as a model selection approach for all combinations of parameters was computationally infeasible. The equilibrium parasite abundance (*θ*) was modeled using the full Sharpe–Schoolfield relation with both upper and lower thresholds and an activation energy that may be positive or negative. If *θ* were regarded as a carrying capacity and solely determined by the parasite’s requirements, it would be expected to be negatively temperature dependent [49]; however, in our model, *θ* represents an equilibrium abundance that is determined by both host and parasite processes and may therefore show positive or negative temperature dependence depending on whether host resistance or parasite processes are more strongly temperature dependent [13]. Both parasite growth rate (*r*) and the Weibull shape parameter (*β*) were described by the modified Sharpe–Schoolfield with only an upper bound. The DT estimates did not show consistent differences between *β*_*U*_ and *β*_*E*_, so we chose to use one *β* parameter described by the same modified Sharpe–Schoolfield function for both exposed and unexposed hosts. Unlike the other rates determining the host–parasite dynamics, mortality tends to peak at extreme rather than intermediate temperatures. However, the typical U-shaped thermal dependence of mean mortality (*μ*) (see S1 Fig) can still be captured using an adapted Sharpe–Schoolfield model due to the inverse relation between mean survival time and mortality rate [16], an approach that we adopted here. Virulence (α) tended to increase slightly with temperature, but high uncertainty in the DT estimates obscured any clear pattern, so we modeled virulence as a temperature-independent parameter. The MTE functions each contain 4–6 hyperparameters, and the MTE model contains 20 free parameters in total compared to 54 free parameters used in the DT model to fit temperatures separately.

We fitted model equations (Eqs 1–3), including the MTE functions for each parameter, via a likelihood function that used as data inputs the time of death for unexposed and exposed individuals as well as the number of parasites per infected individual at the time of death. The probabilities associated with the time of death data were calculated by adapting the model (Eqs 1–3) into a statistical survival model, whereas the probabilities associated with the parasite abundances at the time of death were modeled as a Poisson random variable with expectation equal to the model prediction from Eq 3. The final likelihood function was then the product of Bernoulli and Poisson probabilities associated with the data on time of death and parasite abundance at death. The model was fitted using data cloning [50], a statistical approach that yields unbiased maximum likelihood estimates using Markov chain Monte Carlo (MCMC) and allows for the diagnosis of parameter nonestimability [51], which was a potential concern given the complexity of the metabolic host–parasite model. We implemented data cloning using the MCMC software JAGS [52], interfacing with R [53] via the package dclone [54]. Further details of the likelihood function, model fitting (S2 and S3 Tables), and parameter estimability (S2–S5 Figs) are given in S1 Text.

To analyze the cost of *O*. *colligata* infection to *D*. *magna* in relation to temperature, we calculated the expected lifespan for unexposed and exposed individuals from both the DT and MTE models using the Darth Vader Rule [55], which states that the expected lifespan is equal to the integral of the survival function, which we obtained by discretizing and simulating Eqs 1–3. The expected lifespans were compared for both DT and MTE models graphically, and for the MTE model, we also calculated the percent reduction in lifespan due to infection as a function of temperature. We were not able to apply formal model selection statistics to compare DT and MTE models because parasite growth or abundance parameters in the DT model are not estimable at 6.0 °C and 9.5 °C due to lack of parasite growth and also at 29.7 °C and 33.3 °C because rapid host mortality precludes observation of parasite growth (S1 Text, S4 Table, S3 Fig). Consequently, the MTE model is fitted to a larger dataset than the DT model, which prohibits us from formally comparing these two models using methods such as AIC.

## Supporting information

S1 Table Metabolic models. Equations for the different temperature models applied to parameters in the host–parasite equations (Eqs 1–3).
(XLSX)

S2 Table Priors for DT model. Parameterization of the lognormal priors on the discrete temperature parameters. DT, discrete temperature.
(XLSX)

S3 Table Priors for MTE model. Parameterization of the normal (†) and lognormal priors on the metabolic hyperparameters. MTE, metabolic theory of ecology.
(XLSX)

S4 TableDT model estimates.MLEs and SE for the host–parasite parameters from 15 clones of the data, estimated independently at each temperature. Parameters for which convergence of the MCMC chains could not be achieved are highlighted in yellow ($\hat{R}>1.1$), and parameters that converged but were not estimable are highlighted in red. DT, discrete temperature; MCMC, Markov chain Monte Carlo; MLE, maximum likelihood estimate.(XLSX)

S5 Table Temperature data. Mean and standard deviation of temperature treatments.
(XLSX)

S6 Table Summary of reproduction data. Mean fecundity (offspring produced per d) of individuals in the unexposed and exposed treatments across temperature. The 95% confidence intervals overlap at all temperatures excluding 29.7 °C. Exposed individuals at 29.7 °C appeared to have reduced reproduction during the four-d exposure period, despite having very low levels of infection (Table 1).
(XLSX)

S1 Fig Modified Sharpe–Schoolfield model. Examples of (a) the original Sharpe–Schoolfied model as applied to the shape parameter (*β*), parasite growth rate (*r*), and parasite equilibrium abundance (*θ*) and (b) the modified Sharpe–Schoolfield model as applied to the host mean mortality rate (*μ*). Equations are given in S1 Table. Parameters were the same in both plots: *E* = 0.65 eV, *E*_*H*_ = *E*_*L*_ = 5*E*, *T*_*H*_ = 30 °C, *T*_*L*_ = 12 °C.
(TIFF)

S2 Fig DT model estimability. Estimability diagnostics for discrete temperature parameter estimates, shown as the variance in the posterior divided by the variance in the posterior at one clone, over increasing numbers of clones (from 1 to 15). If parameters are estimable, the scaled variance will approach zero as *K* → ∞ (grey line). If the MCMC algorithm did not converge (open circles), we cannot infer anything about estimability (e.g., *r* at 6.0 and 9.5 °C). DT, discrete temperature; MCMC, Markov chain Monte Carlo.
(TIFF)

S3 Fig DT model estimates and convergence. DT estimates (points) with MTE predictions (lines). Points in green converged and were estimable (shown in main text), points in red converged but were found to not be estimable, and points in yellow did not converge (S4 Table). DT, discrete temperature; MTE, metabolic theory of ecology.
(TIFF)

S4 Fig MTE model estimates over increasing number of clones. Posterior estimates (± one standard error) for 20 hyperparameters in the metabolic model (Table 2) over increasing number of clones from 1 to 25. The grey line shows the prior density (S3 Table). Constant or increasing variance over increasing number of clones indicates parameter estimability problems (e.g., *T*_*Hβ*_). Green points represent convergence, whereas red points did not converge. MTE, metabolic theory of ecology.
(TIFF)

S5 Fig MTE model estimability. Estimability diagnostics for metabolic hyperparameters, shown as the variance in the posterior divided by the variance in the posterior at one clone, over increasing number of clones from 1 to 25. If parameters are estimable, the scaled variance will approach zero as *K* → ∞ (grey line). Convergence of the MCMC was not an issue for any of these hyperparameters. Green points represent convergence, whereas red points did not converge. MTE, metabolic theory of ecology; MCMC, Markov chain Monte Carlo.
(TIFF)

S6 Fig Cumulative reproduction through time for each *Daphnia magna* individual. Red represents exposed individuals, while blue represents unexposed individuals. Each individual’s cumulative reproduction is shown for the duration of its lifespan, with each line ending on the day that that individual died. Since offspring production was quantified twice per wk while mortality was checked daily; in some cases, the last clutch produced by an individual was counted up to three d after that individual was observed to have died. The small number of offspring that were quantified after host death are not shown as part of this time series, though they are included in the reproduction summary statistics in S6 Table. The data used to make this figure can be found in S2 Data.
(TIF)

S1 Data Data on *Daphnia magna* lifespan and infection load. (CSV)

S2 Data Data on *Daphnia magna* survival and reproduction through time. (CSV)

S1 Text Supporting information. (PDF)

## Abbreviations

DT: discrete temperature
MTE: metabolic theory of ecology
