# Supplementary figures and images for: Empirical evidence that metabolic theory describes the temperature dependency of within-host parasite dynamics
Source: PLoS Biol. 2018 Feb 7;16(2):e2004608. doi: 10.1371/journal.pbio.2004608 (PMC5819823; doi:10.1371/journal.pbio.2004608)

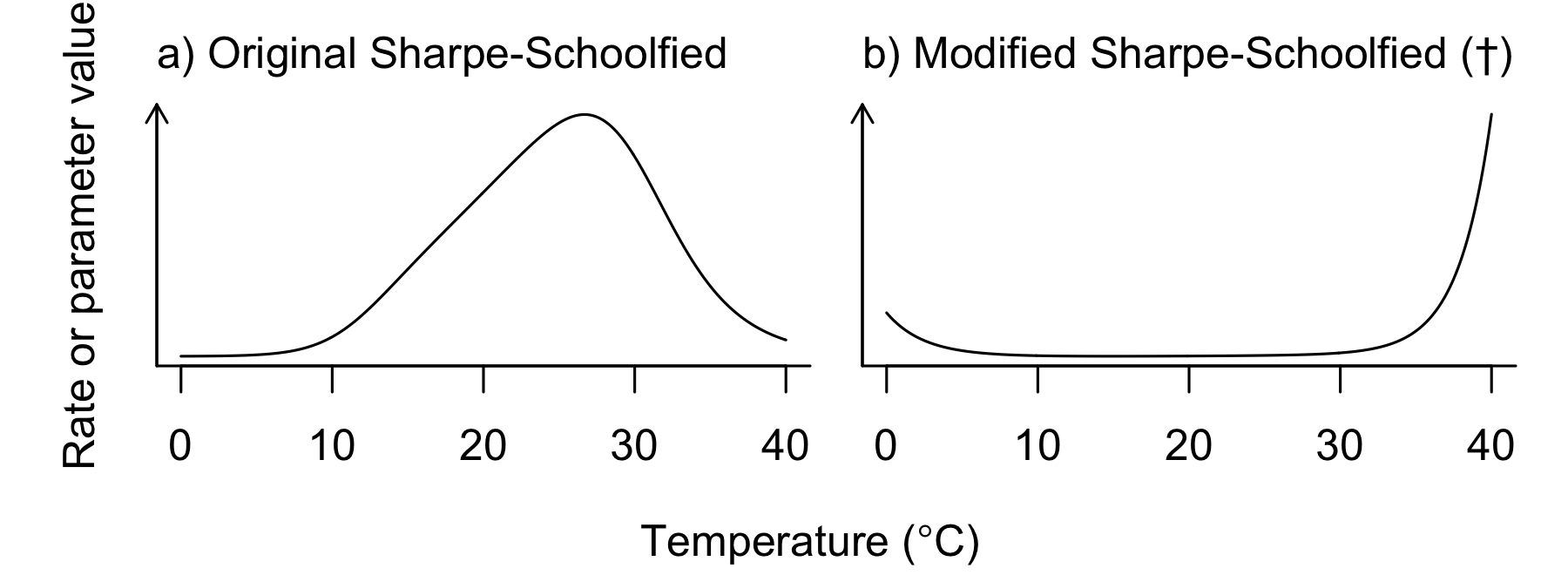

Supplement: S1 Fig — Examples of (a) the original Sharpe–Schoolfied model as applied to the shape parameter (β), parasite growth rate (r), and parasite equilibrium abundance (θ) and (b) the modified Sharpe–Schoolfield model as applied to the host mean mortality rate (μ). Equations are given in S1 Table. Parameters were the same in both plots: E = 0.65 eV, EH = EL = 5E, TH = 30 °C, TL = 12 °C. (TIFF) [file pbio.2004608.s007.tiff]

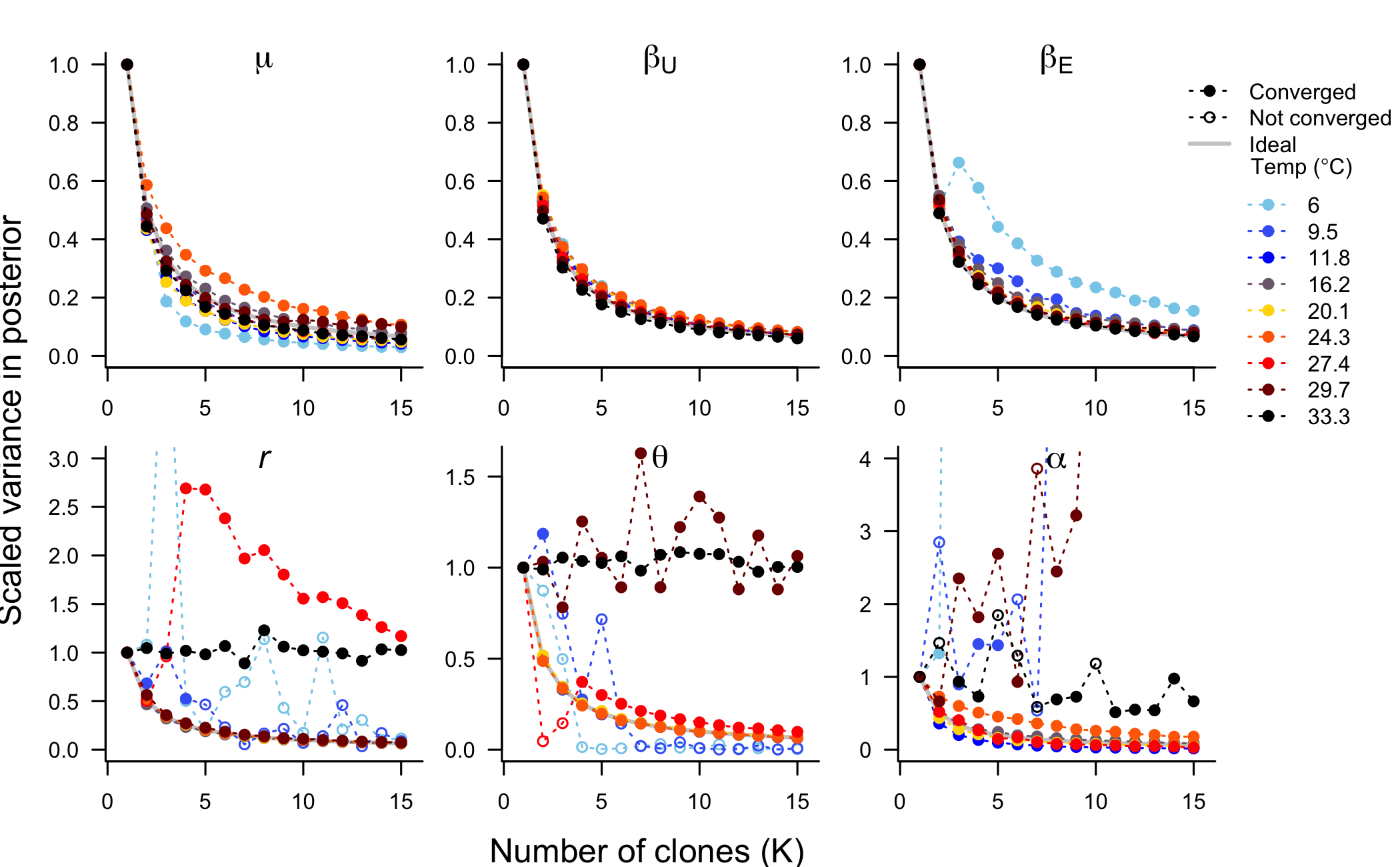

Supplement: S2 Fig — Estimability diagnostics for discrete temperature parameter estimates, shown as the variance in the posterior divided by the variance in the posterior at one clone, over increasing numbers of clones (from 1 to 15). If parameters are estimable, the scaled variance will approach zero as K → ∞ (grey line). If the MCMC algorithm did not converge (open circles), we cannot infer anything about estimability (e.g., r at 6.0 and 9.5 °C). DT, discrete temperature; MCMC, Markov chain Monte Carlo. (TIFF) [file pbio.2004608.s008.tiff]

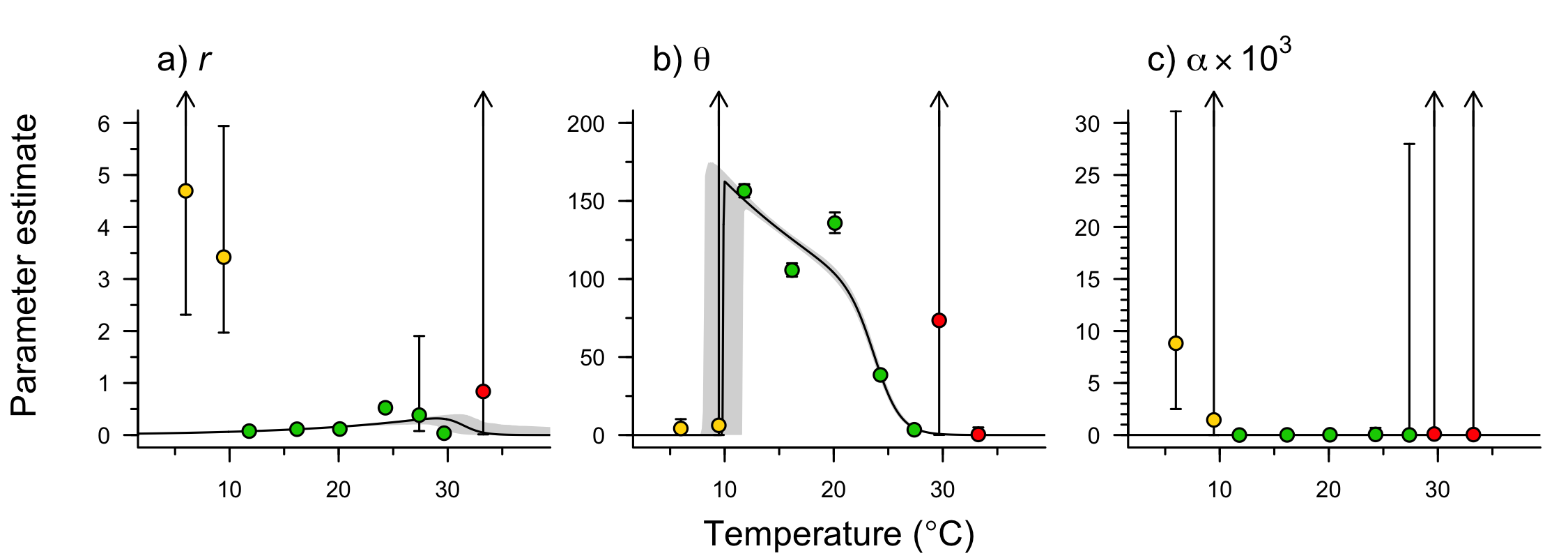

Supplement: S3 Fig — DT estimates (points) with MTE predictions (lines). Points in green converged and were estimable (shown in main text), points in red converged but were found to not be estimable, and points in yellow did not converge (S4 Table). DT, discrete temperature; MTE, metabolic theory of ecology. (TIFF) [file pbio.2004608.s009.tiff]

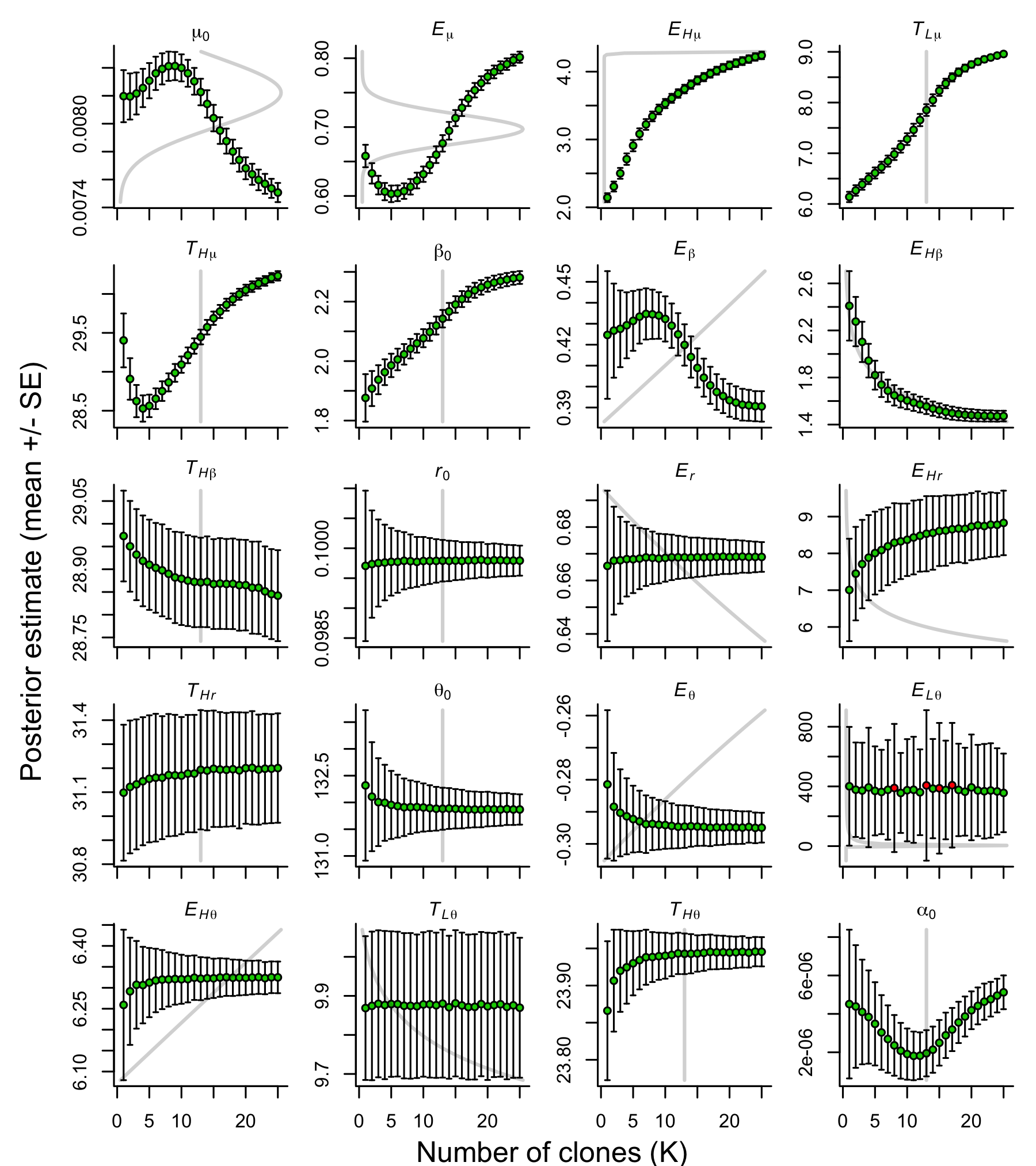

Supplement: S4 Fig — Posterior estimates (± one standard error) for 20 hyperparameters in the metabolic model (Table 2) over increasing number of clones from 1 to 25. The grey line shows the prior density (S3 Table). Constant or increasing variance over increasing number of clones indicates parameter estimability problems (e.g., THβ). Green points represent convergence, whereas red points did not converge. MTE, metabolic theory of ecology. (TIFF) [file pbio.2004608.s010.tiff]

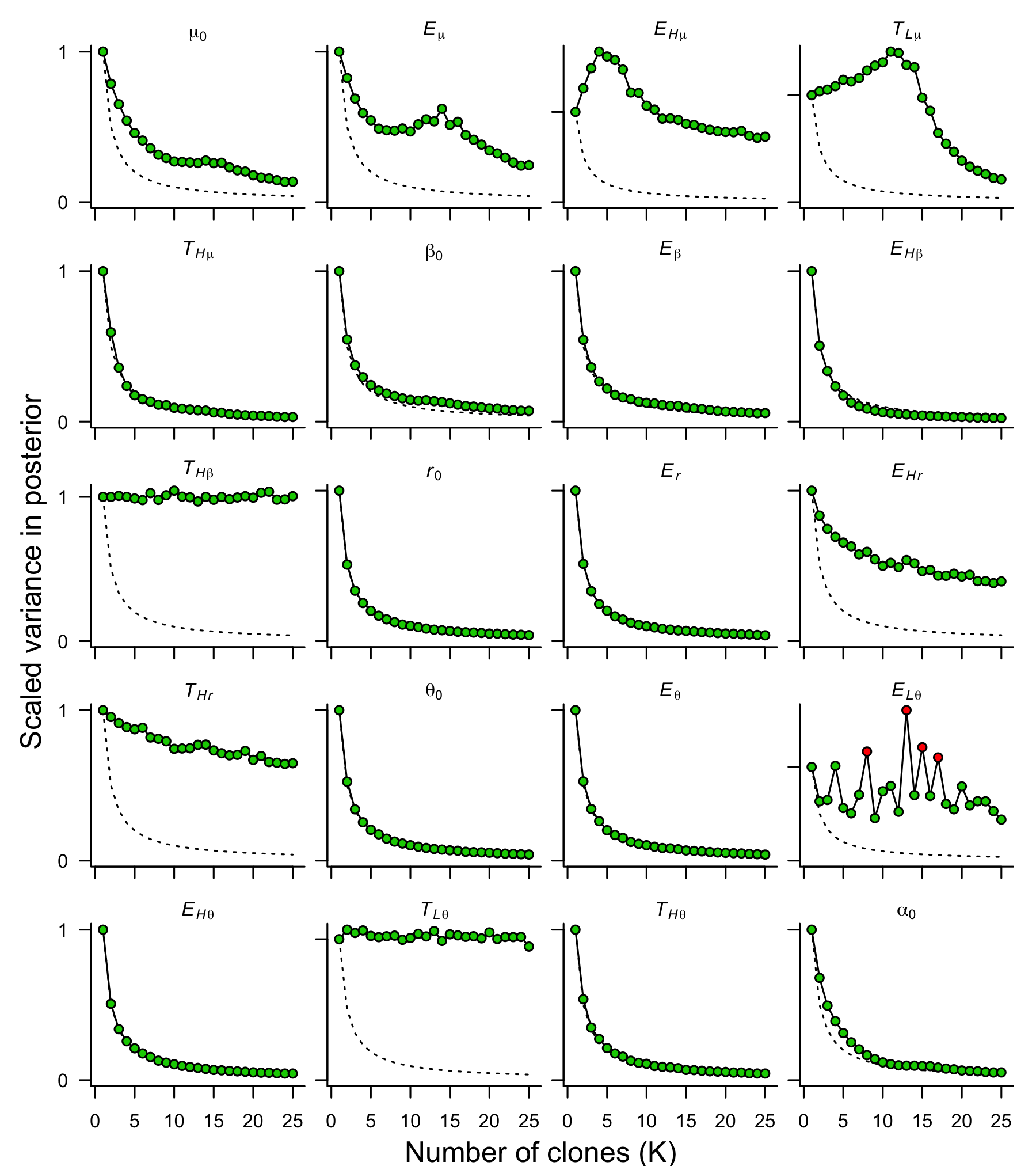

Supplement: S5 Fig — Estimability diagnostics for metabolic hyperparameters, shown as the variance in the posterior divided by the variance in the posterior at one clone, over increasing number of clones from 1 to 25. If parameters are estimable, the scaled variance will approach zero as K → ∞ (grey line). Convergence of the MCMC was not an issue for any of these hyperparameters. Green points represent convergence, whereas red points did not converge. MTE, metabolic theory of ecology; MCMC, Markov chain Monte Carlo. (TIFF) [file pbio.2004608.s011.tiff]

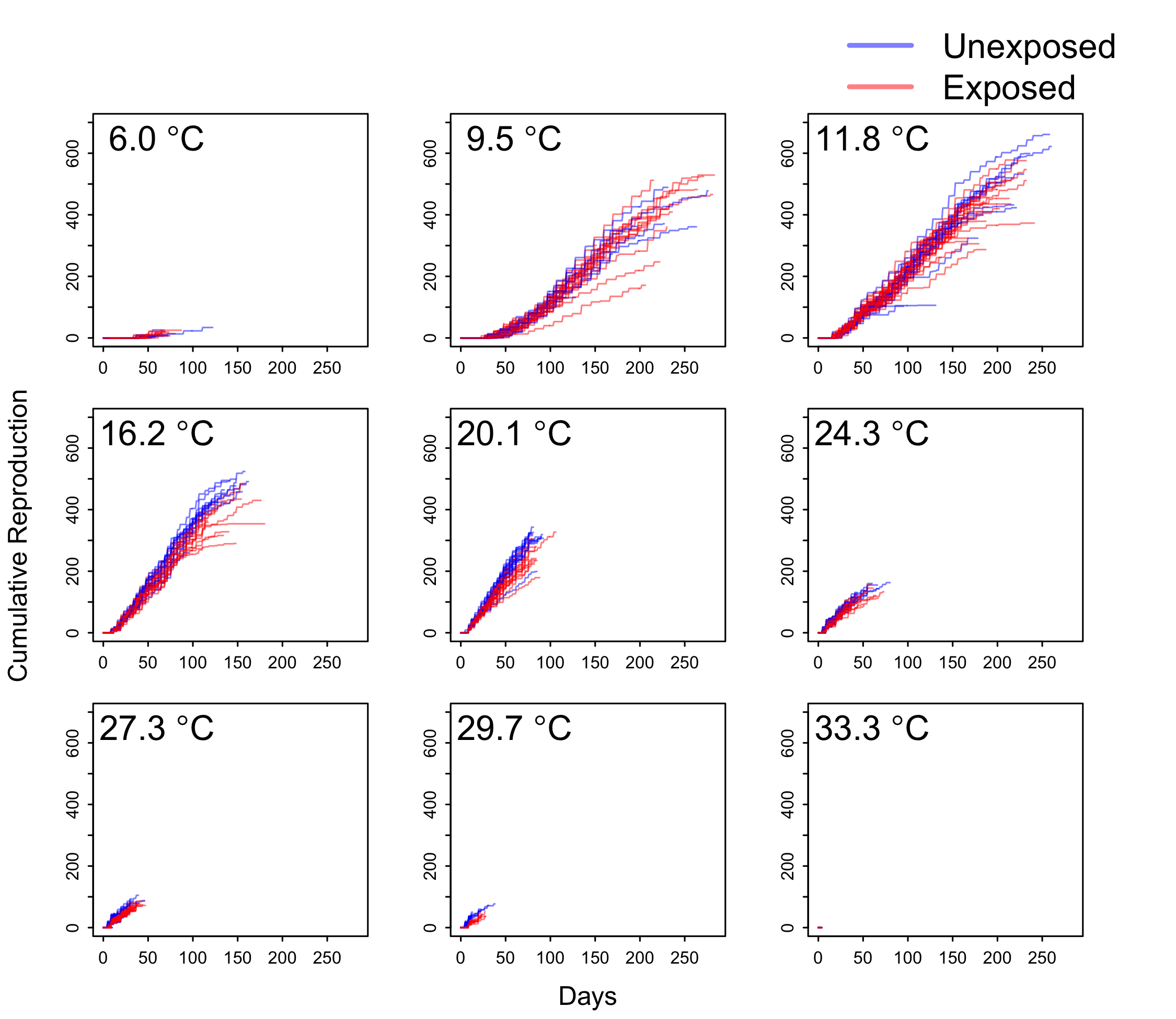

Supplement: S6 Fig — Red represents exposed individuals, while blue represents unexposed individuals. Each individual’s cumulative reproduction is shown for the duration of its lifespan, with each line ending on the day that that individual died. Since offspring production was quantified twice per wk while mortality was checked daily; in some cases, the last clutch produced by an individual was counted up to three d after that individual was observed to have died. The small number of offspring that were quantified after host death are not shown as part of this time series, though they are included in the reproduction summary statistics in S6 Table. The data used to make this figure can be found in S2 Data. (TIF) [file pbio.2004608.s012.tif]
