# Supplementary material for: Empirical evidence that metabolic theory describes the temperature dependency of within-host parasite dynamics
Source: PLoS Biol. 2018 Feb 7;16(2):e2004608. doi: 10.1371/journal.pbio.2004608 (PMC5819823; doi:10.1371/journal.pbio.2004608)
Supplement: S1 Text — (PDF) [file pbio.2004608.s015.pdf]

Full Title: Empirical evidence that metabolic theory describes the temperature dependency of within-host parasite dynamics

Short Title: Metabolic theory and within-host parasite dynamics

Authors: Devin Kirk*[1], Natalie Jones[1,2], Stephanie Peacock[1,3], Jessica Phillips[1,4], Péter K. Molnár[1,5] , Martin Krkošek[1], and Pepijn Luijckx[1,6]

Affiliations: [1] Department of Ecology and Evolutionary Biology, University of Toronto, Toronto, Ontario, Canada, M5S 3G5, [2] Department of Ecology, Behavior and Evolution, University of California San Diego, 9500 Gilman Drive, La Jolla, CA 92093, [3] current address: Biological Sciences, University of Calgary, Calgary, Alberta, Canada T2N 1N4, [4] current address: Department of Zoology, University of Oxford, UK, [5] Department of Biological Sciences, University of Toronto Scarborough, Toronto, Ontario, Canada M1C 1A4, [6] current address: School of Natural Sciences, Zoology, Trinity College Dublin, Dublin 2, Ireland

*corresponding author: devin.kirk@mail.utoronto.ca

TEMPERATURE MODELS

As outlined in the main text, we chose different functions for how the parameters α, β, μ, r and θ might change with temperature based on the discrete temperature (DT) estimates. The models used were the temperature-independent model, Sharpe-Schoolfield with upper threshold, and Sharpe-Schoolfield with both upper and lower thresholds. Each of these functions had different 'hyperparameters', or parameters that went into the functions describing the temperature relationships for the five main model parameters in equations (1-3). The different parameter models and the corresponding hyperparameters are outlined in S1 Table.

We used a variation of the Sharpe-Schoolfield models for the average host mortality parameter, μ. The original Sharpe-Schoolfield relation predicts metabolic rates to peak at intermediate temperatures. This form is suitable for the parasite growth rate (r), parasite equilibrium abundance (θ) and also describes the peak in the shape parameter (β) that we observed from the discrete temperature estimates (S1 Fig). However, we expected that the mean mortality rate, μ, would actually be lowest at intermediate, optimal temperatures and potentially increase at extreme temperatures. Therefore, for the parameter μ, we modified the Sharpe-Schoolfield relation (as done by ref. 1) to describe a U-shaped relationship between these rates and temperature (S1 Fig).

For the Sharpe-Schoolfield relations, we chose a reference temperature of T_0 = 15°C (288.15°K). Other parameters were left as free parameters to be estimated from the data, including the activation energy and upper and/or lower inactivation energies and threshold temperatures for the Sharpe-Schoolfield model (S1 Table) with the exception of the lower inactivation energy for μ – we fixed this parameter at five times the activation energy (following ref. 1) for μ because it was found to be non-estimable from our data (see below).

LIKELIHOOD FUNCTION

To adapt the eqns (1-3 in main text) into a statistical survival analysis of the time of death data, we first note that the proportion of unexposed individuals that survive to time *t* is the solution *U*(*t*), while the proportion dead at time *t* is 1-*U*(t). From basic survival analysis theory, the probability density function of the time of death of an unexposed individual is *d*(1-*U*(t))/*dt*. The data on time of death were resolved at a temporal granularity of individual days (*Daphnia* were checked for deaths once each day), and so the probability of an observed death at time *t* is the probability that an individual died between day *t*-1, when it was observed alive, and day *t*, when it was observed dead:

$$\Pr\big(\text{death in } (t-1,t)\big) = \int_{t-1}^{t} \frac{d\big(1-U(s)\big)}{ds} = U(t-1) - U(t) \qquad \text{(eqn S1)}$$

We applied the same equation for the probability of death for exposed individuals, replacing *U*(t) with *E*(t). The probability of the observed time of death given by eqn S1 was then input into the likelihood function as a Bernoulli probability. The data on parasite abundance at the time of death were incorporated into the likelihood function by modeling the probabilities of those data via a Poisson random variable with expectation equal to the model prediction from eqn 3 for the number of parasites at the time of death exposed individuals. The final likelihood function was then the product of Bernoulli and Poisson probabilities associated with the data on time of death and parasite abundance at death.

MODEL FITTING

Methods

As outlined in the main text, we obtained maximum likelihood estimates for the parameters (or hyperparameters) in the host-parasite model described by equations (1-3) using data cloning [2]. Data cloning is a statistical approach that, as the name implies, clones the data in a Bayesian framework so that the likelihood ‘overwhelms’ the prior, with the benefit of yielding unbiased maximum likelihood estimates using MCMC. In addition, the data cloning approach allows for the diagnosis of parameter non-estimability [3], which was a potential concern given the complexity of our metabolic host-parasite model. Parameter non-estimability may arise due to structural non-identifiability of model parameters due to the form of the model, or a lack of appropriate data with which to estimate parameters. In either case, inference on model parameters that have estimability problems is cautioned against. For further information on parameter estimability and identifiability, we refer interested readers to [2-4] and references therein.

We implemented data cloning using the MCMC software JAGS [5] interfacing with R [6] via the package dclone [7]. These packages make use of the built-in parallelization options for MCMC fits with multiple independent chains. Convergence of independent MCMC chains on the highest posterior density made us confident that we had, indeed, found the maximum likelihood estimate using data cloning. In addition, convergence was a necessary prerequisite to drawing any inference about the estimability of model parameters. If convergence was not achieved, then not

only were we unable to estimate parameter values, but we were unable to even say whether those parameters were estimable or not. We assessed convergence using the Gelman and Rubin's convergence diagnostic ($\widehat{\mathrm{R}}$)[8], where an $\hat{R} < 1.1$ indicated convergence.

For the model fitting procedure, we could not solve eqns (1-3) analytically to obtain model predictions over time, and so within the MCMC simulations, the model was solved numerically using initial conditions $U_0 = 1$, $E_0 = 1$, $P_0 = 1$ to yield the expected survival probability of hosts and number of parasites per host at time t. Note that these initial conditions assume that at the start of the experiments exposed hosts were inoculated with a single parasite and the resulting parasite burden is entirely due to reproduction within the host from that single parasite (as opposed to multiple infections).

The R code used for the analyses is available at: https://github.com/sjpeacock/2018PLoSBiol_MT_host-parasite

Discrete temperature model

For the discrete temperature (DT) model, we estimated the six model parameters - μ, β_U, β_E, r, θ, and α - independently at each of nine temperatures, totaling 54 free parameters. We assumed lognormal priors on each parameter so that they were constrained to positive values (S2 Table). For each temperature, we fitted the model using 1 to 15 clones, assuming the same priors and initial conditions for the MCMC at each number of clones. Fifteen clones appeared sufficient for the posterior to asymptotically approach a value as the number of clones increased (see *Results* below).

For each combination of temperature and number of clones, we ran 10 independent MCMC chains in parallel, which allowed us to assess convergence of the MCMC algorithm. On occasion, several of the chains obviously diverged while the remaining chains converged to biologically reasonable values that were consistent with the other clones; in these cases we removed the chains that did not converge while ensuring at least five chains remained.

We allowed an adaptation phase of 30,000 iterations during which time the samplers adapt their behavior to optimize efficiency of the MCMC algorithm [9] followed by 30,000 iterations for burnin or updating. We then used the subsequent 2000 iterations for inference.

Metabolic model

The model assuming temperature-dependent relationships for each of the six model parameters was fitted to the entire dataset (i.e., all temperatures) simultaneously. We chose the function to describe each parameter based on the shape of the discrete temperature estimates (S1 Table). As outlined in the main text, at first we attempted to use a model selection approach by fitting numerous sub-models for each parameter. However, this quickly became infeasible due to the number of possible sub-model combinations as well as the computational time it took to fit the model using data cloning.

For the metabolic model, we fitted 1 to 25 clones (i.e., more clones than the discrete temperature model) because the posterior parameter estimates were still changing with increasing number of clones at 15 and 20 clones. As for the discrete temperature model, at each number of clones, we

assumed the same prior distribution (S3 Table) and initial conditions, and ran 10 independent MCMC chains. Convergence of the chains was less of a problem for the metabolic model, enabling us to keep at least 7 chains (and usually all 10) for each clone. We used an adaptation phase of 20,000 iterations, a burnin of 20,000 iterations, and used the subsequent 2000 iterations for inference.

Results

Discrete temperature model

Parameters associated with the host equation, namely μ and β, converged and were estimable at all temperatures (S2 Fig). However, parameters for parasite growth (r), parasite equilibrium abundance (θ), and virulence (α) had some problems with either convergence or estimability at the extreme temperatures (S2-3 Fig). This is not surprising given that parasite survival was low to zero at these temperatures (Fig 2 in main text), yielding few data to inform these parameters.

In particular, all three of these parasite parameters did not converge for 6.0°C and 9.5°C where there were no parasites observed. At 33.3°C, the MCMC algorithm converged but examination of the variance in posterior over increasing number of clones suggested that the parasite parameters were not estimable (S2 Fig). Again, there were no parasites observed at 33.3°C, so this is not surprising. Finally, the parasite equilibrium abundance and virulence converged but were not estimable at 29.7°C (S2 Fig). Although parasites were observed at this temperature and parasite growth rate was estimable, hosts did not survive for long, which may have hindered our ability to estimate parasite equilibrium abundance and virulence of associated parasites.

Metabolic model

There were no problems with convergence of the MCMC chains for any of the metabolic hyperparameters, allowing us to assess the estimability of all the metabolic-model hyperparameters. We had to use a higher number of clones (up to 25) compared to the discrete temperature model because some parameter estimates (e.g., μ_0, E_μ) continued to change with increasing number of clones beyond 15 clones (S4 Fig).

As previously mentioned, the lower inactivation energy for μ, $E_{L\mu}$, was not estimable and was a key parameter for predicting the average lifespan of hosts over increasing temperature. As a result, we decided to fix this parameter at five times the activation energy (as in ref. 1) for μ (i.e., $E_{L\mu} = 5 \times E_\mu$) and proceed with estimating other parameters based on this assumption.

Other parameters that were found to be non-estimable given our data were the upper temperature threshold on β ($T_{H\beta}$) and the lower inactivation energy and temperature threshold for the parasite equilibrium abundance ($E_{L\theta}$ and $T_{L\theta}$, respectively)(S5 Fig).

The discrete temperature estimates for β did not show an obvious decrease in β at our upper temperatures, which may explain why $T_{H\beta}$ was not estimable. Further experiments at higher temperatures may allow this parameter to be estimated.

The non-estimability of $E_{L\theta}$and $T_{L\theta}$ may have been due to a lack of parasite data at the lowest temperatures, combined with a sharp transition to relatively high numbers of parasites at 11.8°C.

Additional experiments in between 9.5°C and 11.8°C may capture the transition from $\theta = 0$ to $\theta = 156$ (S4 Table) and help estimate these two parameters.

EXPERIMENTAL METHODS

General

In the months leading up to the experiment, we maintained parasite-free stock populations of *D. magna* in 400mL jars containing artificial *Daphnia* medium (ADaM)[10]. All individuals were asexual clones (Fi-Oer3-3 – Lat: 59.815767, Long: 23.259183**)** kept under standardized lab conditions (mesocosm with ADaM, 16:8 hour light:dark cycle, and fed 3 times per week with the algae *Monoraphidium minutum*). Lines of the clone that were infected with an isolate of their natural microsporidian parasite *Ordospora colligata* were maintained under the same laboratory conditions.

Additional Experimental Details

Mature *D. magna* females were fed *ad libitum* and transferred to new mesocoms (8-10 mature individuals per mesocosm) every 3 days for 3 weeks prior to the initiation of the experiment. We began the experiment by collecting juveniles that were less than 48 hours old from these mesocosms. We used a dissecting microscope to select females based on morphological differences. Female offspring were immediately transferred to 400 ml mesocosms with *ad libitum* algae and a small amount of cetyl alcohol to reduce surface tension (to prevent animals from floating). After 24 hours, individual *Daphnia* were transferred to their own 80 ml mescosm (with 15 million algae and a small amount of cetyl alcohol) and randomly assigned to a temperature and exposure treatment.

When the 648 primary mesocosms were placed into their assigned temperature baths for a 48-hour acclimatization period, we also acclimatized 432 extra *D. magna* in individual mesocosms divided evenly among the treatments that acted as backups in case hosts in the primary mesocosms died during the acclimatization period. Prior to applying any infection treatments, all primary individuals that died during the acclimatization period were replaced with individuals from the extra mesocosms.

Mesocosms were placed in trays which were situated in a water baths controlled by chillers (Coralife® Aquarium Chiller ¼ HP) for the 6.0°C and 9.5°C treatments and by heaters (Blueline™ 1000W Titanium Heating Element and Blueline™ Temperature Controller) in the remaining seven 11.8°C-33.3°C treatments. Water baths were housed in two environmentally controlled chambers (set at 11°C and 21°C) and we used submersible temperature loggers to record water temperatures at 30-minute intervals for the duration of the experiment. Temperature was recorded in each water bath from day 0 until all individuals within that temperature treatment had died. Mean and standard deviation for each temperature treatment can be observed in S5 Table.

On day 0, 15 million *M. minutum* were dispensed into each mesocosm. For the duration of the experiment after day 0, each replicate was fed 30 million *M. minutum* 3 times per week. Every 7 days, *D. magna* were transferred into a new mesocosm with 80mL ADaM and 30 million *M.*

minutum. 4 days after each transfer, offspring were removed from the mesocosm to eliminate any competition for food. To accomplish this, *D. magna* were captured in a pipette, and the medium was poured through a filter to remove offspring. The filter is not small enough to remove spores of *O. colligata*. After removing offspring, *D. magna* were placed back into the mesocosm. The medium in each mesocosm was supplemented with distilled water to the 80mL mark if the medium significantly evaporated during the week.

Parasite Spore Dose and Placebo Dose

The spore solution was created using a mortar and pestle to grind up approximately 2500 adult *D. magna* from nine 4-liter *O. colligata* stock populations. We homogenized the spore mixture, and then quantified spore density using a hemocytometer under 400x phase contrast microscopy. The placebo was made using the same protocol, except that the *D. magna* originated from the uninfected stock populations. We used microscopy to confirm that the placebo stocks were not contaminated with *O. colligata.* First exposure (day 0) occurred when *D. magna* were between 4 and 6 days old. Second, third and fourth exposure occurred on days 1, 2, and 3 respectively. On each exposure day, 1mL of the spore and placebo doses were dispensed into the exposed and control mesocosms respectively. Exposed replicates were dosed with a total of 112,000 spores over the four-day period.

Host Reproduction

We quantified offspring production of each host twice per week, either by counting the number of juveniles remaining in each mesocosm after transferring the focal animal, or by isolating the focal host from its mesocosm (in a glass pipet) and removing the offspring by pouring the contents of the mesocosm over a filter (250 μm) before placing the focal host back in its mesocosm. The data clearly indicate an effect of temperature on host reproduction (S6 Fig, S6 Table), and perhaps a weak effect of the parasite on host reproduction at some temperatures (S6 Fig, S6 Table). As described in the main text, exploring the utility of MTE sub-models for predicting the effects of host reproduction on disease dynamics requires a between-host model of disease dynamics. This was outside the scope of the current study, as our model (eqns. 1-3 in main text) describes parasite dynamics at the within-host level and does not include host reproduction.

REFERENCES

1. Molnár PK, Kutz SJ, Hoar BM, Dobson AP (2013) Metabolic approaches to understanding climate change impacts on seasonal host-macroparasite dynamics. *Ecol. Lett.,* 16:9–21.
2. Lele SR, Dennis B, Lutscher F (2007) Data cloning: easy maximum likelihood estimation for complex ecological models using Bayesian Markov chain Monte Carlo methods. *Ecol. Lett.,* 10:551–563.
3. Lele SR, Nadeem K, Schmuland B (2010) Estimability and likelihood inference for generalized linear mixed models using data cloning. *J. Am. Stat. Assoc.*, 105:1617–1625.
4. Campbell D, Lele S (2014) An ANOVA test for parameter estimability using data cloning with application to statistical inference for dynamic systems. *Comput. Stat. Data Anal.*, 70:257-267.
5. Plummer M (2003) JAGS: A program for analysis of bayesian graphical models using gibbs

sampling. In: (*Proceedings of the 3rd international workshop on distributed statistical computing).* (eds. Hornik. K., Leisch, F., & Zeileis, A.). Vienna, Austria. pp. 1-10.

6. R Core Team (2016) R: a language and environment for statistical computing. Vienna, Austria. http://www.r-project.org/.
7. Sólymos P (2010) dclone: data cloning in R. R J., 2:29–37**.**
8. Gelman A, Rubin DB (1992) Inference from iterative simulation using multiple sequences. *Stat. Science*, 7:457-511.
9. Plummer M (2016) rjags: Bayesian Graphical Models using MCMC. R package version 4-6. https://CRAN.R-project.org/package=rjags
10. Ebert D, Rainey P, Embley TM, Scholz D (1996) Development, life cycle, ultrastructure and phylogenetic position of *Pasteuria ramosa* Metchnikoff 1888: rediscovery of an obligate endoparasite of *Daphnia magna* Straus. *Phil. Trans. R. Soc. B* 351:1689–1701.
